# Categorical phoneme labeling in children with dyslexia does not depend on stimulus duration

**DOI:** 10.1121/1.5116568

**Published:** 2019-07-18

**Authors:** Gabrielle E. O'Brien, Daniel R. McCloy, Jason D. Yeatman

**Affiliations:** Institute for Learning and Brain Sciences, University of Washington, Seattle, Washington 98105, USA

## Abstract

It is established that individuals with dyslexia are less consistent at auditory phoneme categorization than typical readers. One hypothesis attributes these differences in phoneme labeling to differences in auditory cue integration over time, suggesting that the performance of individuals with dyslexia would improve with longer exposure to informative phonetic cues. Here, the relationship between phoneme labeling and reading ability was investigated while manipulating the duration of steady-state auditory information available in a consonant-vowel syllable. Children with dyslexia obtained no more benefit from longer cues than did children with typical reading skills, suggesting that poor task performance is not explained by deficits in temporal integration or temporal sampling.

## I. INTRODUCTION

A popular hypothesis is that dyslexia, a learning disability that affects the development of reading skills, is in fact the result of a subtle impairment in the way speech sounds are processed (Farmer and Klein, 1995; Van Ingelghem *et al.*, 2005; Poelmans *et al.*, 2011; Tallal, 1980). It is well established that both adults and children with dyslexia tend to perform worse than their non-dyslexic peers on phoneme categorization tasks, in that they tend to be less consistent at labeling speech sounds in a stimulus continuum, even for the category exemplar sounds at the continuum endpoints (Brandt and Rosen, 1980; Hakvoort *et al.*, 2016; O'Brien *et al.*, 2018; Serniclaes *et al.*, 2004; Zhang *et al.*, 2012). This yields psychometric models of their performance that exhibit shallower slope than models of typical readers' performance. Across studies employing a variety of experimental paradigms, moderate effect sizes on psychometric slope are typically found (estimated to be 0.66 standard deviations on average by Noordenbos and Serniclaes, 2015), suggesting a reproducible group difference, albeit with considerable overlap between dyslexic and control groups.

It has been posited by several authors that individuals with dyslexia are specifically impaired at temporal processing, which has been argued to affect the processing of brief sounds (Gaab *et al.*, 2007; Lehongre *et al.*, 2011; Richardson *et al.*, 2004; Tallal, 1980) and the processing of rapidly changing acoustic cues like formant transitions (Vandermosten *et al.*, 2010; Vandermosten *et al.*, 2011). There is mixed experimental evidence from auditory psychophysics to support this temporal processing hypothesis: while there is little evidence for impairments in gap detection, significant group differences have been somewhat reliably found for amplitude modulation detection, rise time detection, and duration discrimination (for a meta-analysis, see Hämäläinen *et al.*, 2013). However, the details of these studies are often at odds with one another: while some studies have found differences in slow-rate (<5 Hz) amplitude modulation detection (Rocheron *et al.*, 2002; Stuart *et al.*, 2006), other studies have not (Amitay *et al.*, 2002; Rocheron *et al.*, 2002; Witton *et al.*, 2002). There are similarly contradictory findings for faster modulation rates: while some studies report group differences in detection of modulation rates >100 Hz (Lorenzi *et al.*, 2000; Menell *et al.*, 1999; Rocheron *et al.*, 2002), this is not a consensus finding (Amitay *et al.*, 2002; Stuart *et al.*, 2006). Furthermore, a meta-analysis of the effect sizes associated with psychophysical studies of basic auditory processing in individuals with dyslexia suggested that none of the temporal measures considered—amplitude modulation, duration discrimination, or rise time detection—was associated with more than 52% non-overlap between control and dyslexic groups (Hämäläinen *et al.*, 2013). As such, the literature does not clearly support a specific temporal processing deficit in the majority of individuals with dyslexia.

In previous work, we investigated the possibility that the abnormal performance on phoneme labeling tasks seen in individuals with dyslexia was driven by the presence of dynamic speech cues (O'Brien *et al.*, 2018). We replicated the study design of Vandermosten and colleagues (Vandermosten *et al.*, 2010; Vandermosten *et al.*, 2011), comparing identification performance on two different stimulus continua: one featuring a steady-state spectral envelope cue and the other containing a dynamic formant transition cue. In the Vandermosten group's studies, a cohort of individuals with dyslexia produced shallower psychometric functions (on average) than a control group when asked to label sounds that varied along the formant transition continuum, but not when they labeled sounds on the steady-state continuum. In our study, by contrast, individuals with dyslexia showed reduced ability to categorically label speech sounds along both continua. One potentially important difference between the two studies is that in ours, the duration of the informative cues was equated (i.e., the formant transition and spectral envelope cue were both present for 100 ms), whereas in Vandermosten and colleagues' original studies they were not (i.e., the formant transition lasted 100 ms and the spectral envelope cue was present for 350 ms).

Our results indicated that individuals with dyslexia are not specifically impaired at categorizing speech sounds on the basis of dynamic auditory information—when presented with brief fricatives that differed based only on spectral envelope, our participants with dyslexia showed a similar degree of impairment as when they were labeling stop consonants. However, the discrepancy between the results of our study and Vandermosten and colleagues' results for steady-state cues raised the possibility that many individuals with dyslexia have impaired categorization on the basis of *any* “brief” cue. This would be consistent with the hypothesis of Tallal and colleagues, who proposed that individuals with dyslexia perform poorly whenever rapid auditory processing is involved (Merzenich *et al.*, 1996; Tallal *et al.*, 1996). It would also be consistent with the hypothesis of Goswami, who proposed that individuals with dyslexia irregularly sample auditory information (Goswami, 2011). If insufficient sampling were the primary problem, then allowing a listener to acquire more samples by providing a longer exposure to the cue should reduce the performance gap between individuals with dyslexia and typical readers. Agnostic of these two temporal processing hypotheses, it is also conceivable that some participants with dyslexia weigh sensory information differently than their typically developing peers (Nittrouer, 1999), and require more evidence acquired over more “looks” at a signal to make consistent category judgments.

To investigate these possibilities, we sought to determine whether longer exposure to steady-state acoustic cues in the context of a phoneme identification task benefits children with dyslexia more than children with typical reading skills. Note that the definition of dyslexia varies throughout the literature, and the majority of studies on auditory processing in this population employ a group-level design (Calcus *et al.*, 2016; Law *et al.*, 2014; Ramus *et al.*, 2003; Talcott *et al.*, 2000). Typically, a sample of individuals with low standardized reading scores and/or a diagnosis of dyslexia are compared to a sample of individuals with average or better standardized reading scores, yielding two groups that are well separated in terms of reading skill. While we wish to maintain parity with existing studies for ease of comparison, we believe that treating reading skill as a continuous variable provides a more insightful analysis for several reasons. First, previous work (including our own) suggests that the cutoff between individuals with dyslexia and below-average readers is arbitrary (O'Brien *et al.*, 2018; Shaywitz *et al.*, 1992). Second, several auditory measures, including measures of categorical labeling, have been shown to vary continuously across a range of reading skills (Goswami *et al.*, 2002; O'Brien *et al.*, 2018; Vandermosten *et al.*, 2010). As such, we present two kinds of analyses in the present study: first, we examine how measures of auditory perception are related to reading skill as a continuous variable, including readers who score below-average on literacy assessments but do not meet our criteria for inclusion in the dyslexia group. Second, for continuity with previous studies, we compare individuals with dyslexia (defined as reading scores more than 1 standard deviation below the population mean) with a well-separated control group exhibiting reading skills at or above the population mean.

As in our earlier study, we employed a fricative continuum that spans from [s] to [ʃ]. We tested two conditions: one in which the steady-state fricative is available to listeners for 100 ms (short cue duration), and one in which it is present for 300 ms (long cue duration). We chose these durations because our previous results indicated that participants with dyslexia had difficulty categorizing 100-ms-long fricatives, while Vandermosten and colleagues had shown that participants with dyslexia had no difficulty categorizing 350-ms-long steady-state vowels. As before, we recruited children with a range of reading abilities to participate.

## II. METHODS

### A. Participants

A total of 83 native English-speaking children ages 8–12 years were recruited for the study. Children without known auditory disorders were recruited from a database of volunteers in the Seattle area (University of Washington Reading and Dyslexia Research Database^1^). Parents and/or legal guardians of all participants provided written informed consent under a protocol that was approved by the University of Washington Institutional Review Board. All subjects demonstrated normal or corrected-to-normal vision. Participants were tested on a battery of cognitive and literacy assessments, including the Woodcock-Johnson IV (WJ-IV) letter-word identification and word attack sub-tests (Schrank *et al.*, 2014), the Test of Word Reading Efficiency (TOWRE; Torgesen *et al.*, 2011), and the Wechsler Abbreviated Scale of Intelligence (WASI-II; Wechsler, 2011). All participants underwent a hearing screening to ensure pure tone detection at octave frequencies between 500 and 8000 Hz in both ears at 25 dB hearing level (HL) or better.

One subject who was initially recruited did not pass the hearing screening and was not entered in the study, and seven others did not meet the inclusion criterion for intelligence quotient (IQ; performance no less than 2 standard deviations below the population mean on the age-normed WASI-II FS-2 and nonverbal IQ measures). One subject was unable to complete training for the experimental task and so did not participate further. Another two subjects yielded data that could not be well fit with a psychometric function and were excluded from further analysis on the basis of low confidence in the psychometric parameter estimates, thus, yielding data from 72 children in total. The guidelines for exclusion are described in greater detail in Secs. II B–II G.

### B. Demographics

In order to understand the relationship between phoneme categorization ability and reading ability, we selected our cohort of participants to span the continuum from impaired to highly skilled readers. Although we treat reading ability as a continuous measure in our statistical analyses, for the purpose of recruitment and data visualization we defined three groups based on the composite Woodcock-Johnson basic reading score (WJ-BRS) and the TOWRE index. For many of our subjects, standardized reading scores from multiple recent visits (within the past 14 months) to the laboratory were available. None of these children had participated in reading interventions or specialized training programs, so in order to gain the most stable measures of reading skill we averaged over available scores. As both the WJ-BRS and the TOWRE index are scored on the same standardized scale, a composite reading skill measure was created by averaging the two metrics for each participant. Using a composite of both measures as our criterion improved the confidence of our group assignments since they are highly correlated measures (*r* = 0.91, *p* < 1e-15). The “dyslexic” group comprised participants whose reading score fell 1 standard deviation or more below the mean (standardized score of 100); above average readers were defined as those with scores above the population mean.

Several individuals fell between these categories, and we have labeled them as “below average” in our dataset. These category delineations are consistent with our previous work (O'Brien *et al.*, 2018) and similar to the definitions used by others (e.g., Rimrodt *et al.*, 2010; Shaywitz *et al.*, 2002). While we have included the complete data for these individuals in our online data release, we opted to focus our statistical group comparisons on the dyslexic and above average groups for ease of comparison with the broader dyslexia literature, where it is typical to compare two well-separated groups. Of the 17 individuals who fell into the below average group, 9 had parental reports of a dyslexia diagnosis. Thus, this group largely consists of individuals who at one point may have had reading difficulties, but have since received sufficient remediation to reach an age-typical reading level.

There were 36 subjects in the dyslexic group (17 male), 17 in the below average group (8 male), and 19 in the above average group (9 male). There were no significant differences in age between groups [Kruskal-Wallis rank sum test, *H*(2) = 2.28, *p* = 0.32], nor was there a significant correlation between age and reading score (*r* = −0.009, *p* = 0.57). We did not exclude participants with Attention Deficit/Hyperactivity Disorder (ADHD) diagnoses from the study because ADHD is highly comorbid with dyslexia (Germanò *et al.*, 2010). We expect this inclusion leads to a more representative sample of children with dyslexia. However, we did account for the presence of ADHD diagnosis in our statistical models. Of our 72 participants, 20 had a formal diagnosis of ADHD: 4 in the above average group, 5 in the below average group, and 11 in the dyslexic group. The difference in prevalence of ADHD across groups was not significant [Kruskal-Wallis rank sum test, *H*(2) = 0.71, *p* = 0.70].

Table I shows group comparisons on measures of reading and cognitive skills. We observed that IQ, both full-scale (incorporating verbal IQ) and nonverbal measures, varied as a function of group. Importantly, by design no subjects had a full-scale or nonverbal IQ falling more than 2 standard deviations below the population mean as measured by the WASI-II. Therefore, although there was a significant difference in IQ scores across groups, we were not concerned that abnormally low cognitive ability would prevent any child from performing the experimental task. To be certain our results were not confounded by this difference, we also included nonverbal IQ as a covariate in our statistical analyses to confirm the specificity of the relationship with reading skills as opposed to IQ.

**TABLE I.** Summary statistics and group differences on various demographic, reading, and cognitive measures. Summary statistics show means and sample standard deviations. Significance assessed by the Wilcoxon rank-sum test. Stars (*) indicate *p*-values that meet threshold for significance (α = 0.05) after Bonferroni correction for 24 comparisons.

	Dyslexic (36 subjects)	Below average (17 subjects)	Above average (19 subjects)	Significance (dyslexic versus below average)	Significance (dyslexic versus above average)
Males/females	17/19	8/9	9/10	—	—
Age	9.7 (1.3)	9.5 (1.2)	10.3 (1.7)	0.962	0.172
WASI-II					
FS-2	96.0 (9.5)	106.4 (14.5)	121.3 (16.2)	0.003	7.423e-7*
Nonverbal IQ	46.5 (6.4)	52.0 (8.0)	60.0 (11.8)	0.023	6.708e-5*
WJ- IV					
WJ-BRS	77.5 (10.6)	95.5 (3.5)	113.5 (9.4)	1.400e-8*	1.484e-9*
Word reading	74.3 (12.0)	94.3 (5.4)	113.1 (10.5)	2.557e-8*	1.463e-9*
Nonword reading	83.2 (10.8)	97.5 (6.5)	112.0 (9.2)	4.692-6*	1.742e-9*
TOWRE 2					
TOWRE index	68.8 (8.3)	91.7 (7.0)	108.6 (9.4)	1.556e-8*	1.465e-9*
Word reading	69.9 (10.7)	93.7 (9.4)	111.8 (11.8)	2.235e-7*	1.456e-9*
Nonword reading	70.9 (7.0)	90.4 (5.7)	104.5 (7.8)	7.218e-9*	1.477e-9*
CTOPP 2					
Phonological awareness	84.2 (11.3)	92.2 (12.8)	102.2 (14.2)	0.035	3.600e-5*
Phonological memory	83.0 (11.8)	91.2 (13.8)	99.6 (19.8)	0.0628	0.001*
Rapid symbolic processing	78.6 (10.3)	92.4 (10.0)	97.5 (13.6)	2.043e-5*	1.321e-5*

### C. Stimuli

Two seven-step speech continua were created using Praat version 6.0.37 (Boersma and Weenink, 2019). Both were /ʃa/∼/sa/ continua, with the sole difference being the duration of the initial fricative. The fricative duration was either 100 or 300 ms. This choice of continuum was motivated by our previous study (O'Brien *et al.*, 2018), in which participants with dyslexia behaved less categorically than above average readers when labeling /ba/∼/da/ and /ʃa/∼/sa/ continua with 100-ms-long consonant cues. In order to test the hypothesis that increased cue duration would assist our dyslexic participants in the labeling task (as predicted by the temporal processing deficit hypothesis), it was important to include at least one condition in which we expected dyslexic participants to behave less categorically than control participants, so we replicated the 100 ms /ʃa/∼/sa/ condition from our prior work, and introduced a 300 ms condition as well.

The /ʃa/∼/sa/ continua were created by splicing synthesized fricatives onto a natural /a/ token excised from a spoken /sa/ syllable. The duration of /a/ was scaled to 250 ms using Praat's implementation of the Pitch-Synchronous Overlap-and-Add (PSOLA) algorithm. Synthesized fricatives contained three spectral peaks centered at 2500, 3500, and 6500 Hz. The bandwidths and amplitudes of the spectral peaks were linearly interpolated between continuum endpoints in seven steps, and the resulting spectra were used to filter white noise. To improve the naturalness of the synthesized fricatives, a gentle cosine on- and off-ramp was imposed on the fricative envelope. The on-ramp lasted 75 ms in the short fricative and 225 ms in the long fricative, and the off-ramp lasted 20 ms in the short fricative and 60 ms in the long fricative. As such, the duration of the ramps was scaled proportional to the entire duration of the fricative with the rise occurring in the first 75% of the fricative and the fall in the last 20%. Aside from this onset/offset ramping (which was applied equally to all continuum steps), the contrastive cue (the amplitudes and bandwidths of the spectral peaks) was steady throughout the duration of each fricative. Stimuli used in the experiment are available online.^2^

### D. Procedure

Stimulus presentation and participant response collection were managed with PsychToolbox for matlab (Brainard, 1997; The Mathworks Inc., 2016). Auditory stimuli were presented at 75 dB sound pressure level (SPL) via circumaural headphones (Sennheiser HD 600). Children were trained to associate sounds from the two speech continua with animal cartoon characters (pink and purple snakes) on the right and left sides of the screen, respectively, and to indicate their answers with right or left arrow keypresses. Throughout all blocks, each cartoon was always associated with the same stimulus endpoint. After every 35 stimulus presentations, a reminder was displayed illustrating the snake associated with each sound.

Practice rounds were administered before the first test block. In practice rounds, participants were asked to categorize only endpoint stimuli and were given feedback on every trial. Participants had to score at least 75% correct on the practice round to advance to the experiment and were allowed to repeat the practice blocks up to three times. As mentioned, one child from the initial recruitment (belonging to the below average group) did not meet this criterion and was not included in the study.

In each test block, participants heard a single syllable and decided which category it belonged to by selecting an animal (no text labels were used). Generally, this was not difficult for our participants. Three subjects (one above average and two dyslexic) lost track of the animals associated with the endpoints after succeeding at the practice rounds. In these cases, the experimenter provided written labels taped to the computer monitor to assist. Each block contained 5 presentations of each step on the continuum for a total of 35 randomly ordered trials.

Six blocks were administered in total. In each block, only stimuli with short (100 ms) or long (300 ms) fricatives were presented (i.e., the stimulus duration was alternated on each block). The order of presentation was counterbalanced across participants such that half began with the 100 ms fricative continuum and half began with the 300 ms fricative continuum.

### E. Psychometric curve fitting

Modeling of response data was performed with Psignifit 4.0, a matlab toolbox that implements Bayesian inference to fit psychometric functions (Schütt *et al.*, 2015). We fit a logistic curve with four parameters modeling the upper and lower asymptotes, the width of the logistic function, and the threshold. The width of the logistic function was transformed to the slope at the threshold value to give a measure of psychometric function slope.

In the Bayesian framework, each of these four parameters is estimated based on experimental observations weighted by a prior distribution. A prior distribution, or “prior,” defines the range over which a parameter could potentially vary. Incorporating this type of prior expectation into the model fitting procedure yields more stable estimates of model parameters, particularly in the case where parameters can be correlated (i.e., slope and asymptote). However, in the case of fitting sigmoidal functions, it is important not to use overly broad or narrow priors for the two asymptotic parameters, as this may lead to biases in slope estimates (Schütt *et al.*, 2015).

To determine the most appropriate priors for fitting the two asymptotic parameters, we utilized a cross-validation approach previously demonstrated in O'Brien *et al.* (2018). Rather than search for the best choice of priors for each asymptote separately, we assumed that the two asymptotes are determined by a common *lapse rate*. A lapse rate of 10% means that the lower and upper asymptotic parameters will be 0.1 and 0.9, respectively. Four-parameter fits of each experimental block were performed with each of seven possible priors: a prior that fixed the lapse rate at zero (in line with the approach in most previous studies of auditory processing in dyslexia), and six uniform distribution priors with lower bounds of zero and upper bounds ranging from 5% to 30% in steps of 5%. Next, the optimal prior was chosen using tenfold cross-validation. For each psychometric function measured for each participant in each condition (long and short fricative), psychometric curves were fit to 90% of the data, and then the summed log likelihood of the held-out 10% of data points was calculated. This process was repeated ten times, once for each unique 10% of the data for each of the possible prior widths. The estimated likelihoods of the held-out data points were used as a goodness-of-fit metric and pooled across blocks and cross-validation runs to determine the median likelihood for each prior width. The goodness-of-fit metric was normalized for comparison across participants by subtracting out each individual's median likelihood with a two-parameter model. Using the standard error rule (selecting the most restrictive prior width at which the standard error does not encompass 0), the optimal prior was determined to be a maximum lapse rate of 15%. As expected, the psychometric fits had the poorest fit to the data when the asymptotes were fixed at 0 and 1 (as in the two-parameter model).

Once we determined the optimal prior for the asymptote parameters, models were re-fit on the full dataset for each combination of participant and cue continuum to obtain final estimates of the four parameters. Any cases where the best-fit threshold parameter were not in the range of the stimulus continuum steps (1–7) were excluded from further analysis. Additionally, one subject in the dyslexic group performed the labeling consistently backwards and produced a sharp psychometric function in the reverse direction, despite repeated reminders about the sound associated with each animal throughout the experiment. The psychometric functions were particularly ill-fitting for this subject and were removed from further analysis. Once the psychometric functions had been fit, data from 72 subjects were available for statistical analysis.

### F. Statistical analysis of parameter estimates

After we fit psychometric functions for each subject in each condition, we used a series of generalized linear mixed models to determine the relationship between reading ability, stimulus duration, and three dependent measures. The first dependent measure was the psychometric slope. The second dependent measure was the average offset of the upper and lower asymptotes of the function (i.e., their deviations from 0 or 1, respectively). For the third dependent measure, we were interested in a composite measure of psychometric function shape incorporating all four of its parameters (slope, threshold, upper asymptote, and lower asymptote). We derived this composite measure by performing principal components analysis on these parameters for each psychometric function collected in the study, transforming each psychometric function to a single variable according to the linear weights prescribed by the first principal component (PC1).

We were motivated to consider the PC1 measure of psychometric function shape because of a well-known challenge in interpreting psychometric function parameters: it is difficult (if not impossible) to fit both the slope and asymptotic parameters without incurring bias in one domain due to these parameters' trading relationship in the optimization space of sigmoidal functions (Treutwein and Strasburger, 1999). Moreover, data that are best fit with non-zero asymptotes are often treated as indicative of lapses of attention, but in fact may (in part) reflect aspects of the deficit under study (O'Brien *et al.*, 2018; Serniclaes *et al.*, 2004). Therefore, ideally, we would model slope and asymptotes simultaneously using a single measure of the psychometric shape that takes all parameters into account—namely, the PC1. We have previously shown this measure to be an effective predictor of reading ability and potentially a more sensitive probe than either slope or asymptotic parameters alone (O'Brien *et al.*, 2018).

For each dependent measure (slope, asymptote, and PC1), fixed-effect predictors with sum coding were used for the continuum (short versus long fricative duration) variable (i.e., the categorical predictors were represented as 0.5 and −0.5). Reading ability (the average of the WJ-BRS and TOWRE index) was entered as a continuous fixed-effect predictor except where otherwise stated. Additional predictors were added for presence/absence of ADHD diagnosis (treatment coding) and nonverbal IQ (WASI-III matrix reasoning score; continuous predictor). A random intercept for participant was also included. For all model analyses, we began with a fully specified model of reading score as a function of the parameters of interest (slope, asymptote, or PC1) plus the two covariates (ADHD and nonverbal IQ) and a random intercept for subject identity. All model fitting was done using the lme4 library for *R* (Bates *et al.*, 2015). The contributions of the covariates were first tested using parametric bootstrapping using the pbkrtest library for *R* (Halekoh and Højsgaard, 2014), which is robust to non-normally distributed residuals. Model terms were retained if the bootstrapped *p*-value of the coefficient being nonzero was less than 0.1. In all cases, the covariates failed this test and were dropped from the model. After testing the covariates, we tested the terms of interest for the study (duration, reading ability, and the interaction between them) using the same approach.

### G. Data availability statement

The de-identified datasets reported and analyzed in the current study, as well as the code for stimulus presentation, data analysis, and figure generation, are available online.^3^

## III. RESULTS

### A. Relationship between phoneme categorization and reading skill

**FIG. 1.**
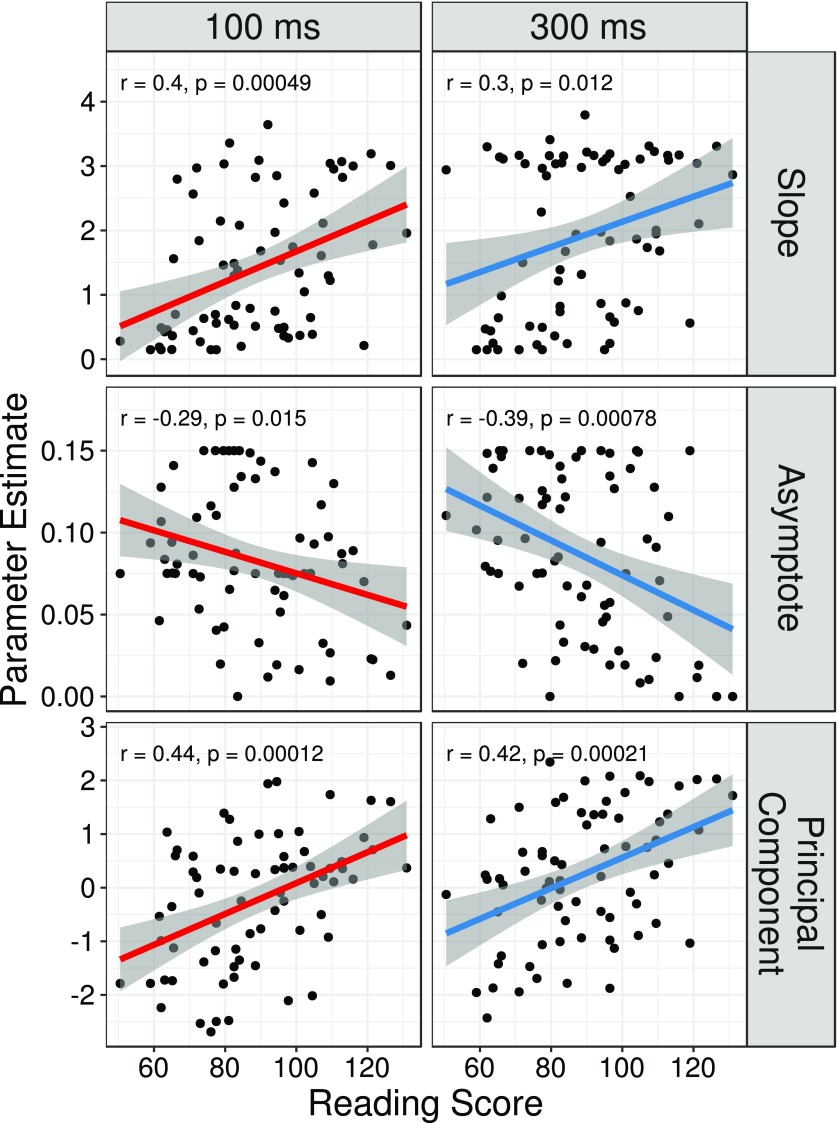
(Color online) Plots of model parameter estimates versus reading score. Each point corresponds to parameter estimates for one subject in one condition (100 ms or 300 ms fricative duration). Lines indicate the best fit regression line with 95% confidence intervals in shaded regions.

We found an association between reading skill and several aspects of psychometric function shape, shown in Fig. 1. Slope, asymptote, and individual PC1 measures are all shown with their correlations to reading ability. Two columns show how these psychometric function parameters are correlated with reading skill at each fricative duration, 100 ms or 300 ms. Upon visual inspection, it is clear that the relationship between task performance and reading skill is largely independent of fricative duration; we confirmed this with generalized linear mixed model analysis. First, we examined the relationship between reading ability and psychometric slope. After model selection, the most parsimonious model of psychometric slope contained a continuous predictor for reading ability (average reading score on WJ and TOWRE), a main effect of stimulus duration, and a random intercept for each participant (see Table II). The interaction between reading score and stimulus duration was not significant and did not survive the model selection procedure. Reading score and stimulus duration were both significant predictors of psychometric slope.

**TABLE II.** The results of model selection for three dependent variables: psychometric function slope, asymptote, and PC1.

Model	Parameter	β	SE	*p*
Slope	Reading score	0.027	0.006	3.132e-5
	Duration (long)	0.509	0.157	0.002
Asymptote	Reading score	−0.0008	0.0002	0.002
PC1	Reading score	0.271	0.006	3.178e-5
	Duration (long)	0.509	0.157	0.0002

We next considered the asymptote as the dependent variable. The same initial model specification and simplification procedure used in the model predicting psychometric slope was also used for the model predicting the average asymptote parameter. The final model of asymptote contained a significant predictor for reading ability (see Table II); there was no evidence for a significant main effect of duration or an interaction between duration and reading ability. Higher reading ability was associated with smaller asymptotic parameters (about 0.08% smaller per 1-unit increase of reading score). Thus, compared to strong readers, poor readers showed shallower psychometric slopes, and were also more likely to label stimuli inconsistently even near the continuum endpoints. On average, subjects showed a steeper psychometric function when provided a longer cue irrespective of their reading ability. There was no evidence to suggest that the degree of within-category consistency (asymptote) depended on the cue duration (short or long fricative).

Finally, we investigated how the PC1 composite measure of psychometric function shape varied with reading skill and stimulus duration. This component explained 37.8% of the variance in our psychometric function data, reflecting a linear combination of the slope, threshold, and both asymptotes (slope, 0.543; threshold, 0.542; lower asymptote, −0.636; upper asymptote, −0.086). The most parsimonious generalized linear mixed model of PC1 contained predictors for reading skill and fricative duration, but not the interaction between these two variables (see Table II). Thus, reading skill and fricative duration were significant predictors of psychometric function shape as summarized by PC1.

While our model selection procedure led us to drop nonverbal IQ from all our models so far, the fact that nonverbal IQ differed significantly among reading groups suggested a need for caution in discarding this covariate. To be sure that nonverbal IQ was not a meaningful predictor of task performance separate from reading skill, we modeled psychometric function shape as a function of nonverbal IQ, now with reading as the covariate. Here, we used PC1 as the outcome measure because PC1 yielded stronger correlations with reading skill than either slope or asymptote measures (see Fig. 1). In this model, nonverbal IQ was not a significant predictor of PC1 [β = 0.018, standard error (SE) = 0.013, *p* = 0.177], but reading skill was (β = 0.023, SE = 0.008, *p* = 0.004). This finding, combined with the outcome of our model selection process, suggests that the observed relationship between reading skill and phoneme categorization task performance could not be explained in terms of our nonverbal IQ measure.

### B. Group differences in phoneme categorization

As discussed in the Introduction, it is conventional in dyslexia research to analyze group-level differences in outcome measures. Therefore, to supplement the findings relating reading score to phoneme categorization, we also conduct a series of group comparisons in order to compare effect sizes to the broader literature on phoneme labeling in people with dyslexia. Because we wish to provide a comparison of two groups that are clearly separated in terms of reading ability, these analyses were carried out using data from our subjects in the dyslexic and above average reader groups (omitting the “below average” group; see Table I).

Using a linear mixed effects model with group as a categorical predictor (above average as the reference group), subject as a random factor, and following the same model selection procedure as before, a model containing main effects of group and duration was selected. In this reduced model, mixed-effect analysis of variance (ANOVA) tests indicated that both main effects were significant predictors of slope [group: *F*(1,50.79) = 7.26, *p* = 0.010, degrees of freedom estimated via the Kenward-Rogers approximation; duration: *F*(1,65.85) = 14.96, *p* < 0.001]. The interaction between duration and group was eliminated by model selection. The estimated Cohen's *d* for the separation of slope by group was 0.78, with a 95% confidence interval ranging from 0.19 to 1.38.

Next, we considered a model of average psychometric asymptote parameter. The most parsimonious model contained only a main effect of group, which was significant [*F*(1, 51.04) = 6.54, *p* = 0.014]. Group separability by lapse rate was measured with Cohen's *d*, giving an effect size of 0.73 with a 95% confidence interval ranging from 0.14 to 1.32.

Last, we confirmed the relationship between group and psychometric PC1 [*F*(1,50.35) = 15.13, *p* < 0.001]. The selected model also included a significant main effect of duration [*F*(1,51.71) = 11.07, *p* = 0.001]. A candidate model including the interaction between duration and group indicated that this term was not significant [*F*(1,66.78) = 0.13, *p* = 0.725]. To assess the separability of the groups, we calculated Cohen's *d* for a group comparison where each individual's PC1 estimate is averaged across the two test conditions (combining long and short durations). There was a large effect size for the comparison between the dyslexic and above average groups (*d* = 1.1, with the 95% confidence interval spanning from 0.50 to 1.72).

To maintain continuity with the broader dyslexia literature, we were primarily interested in comparing two well-separated groups of readers. However, for completeness, we confirmed that the main findings were not substantially altered by including the cohort of below average readers as a third comparison group. Mixed-effect ANOVAs showed that for slope, main effects of group and duration were significant [group: *F*(2,64.98) = 3.53, *p* = 0.035; duration: *F*(1,77.32) = 20.65, *p* < 0.001]. For asymptote, there was a significant main effect only of group [*F*(2,65.23) = 3.73, *p* = 0.029]. For PC1, both group and duration were significant predictors [group: *F*(2,65.67) = 8.48, *p* < 0.001; duration: 5*F*(1,66.53) = 10.38, *p* = 0.002]. All of these results are consistent with those of the two-group analyses.

## IV. DISCUSSION

Our results replicate and extend previous findings about the nature of phoneme categorization deficits in struggling readers. We confirm that reading ability is moderately predictive of the extent to which a child applies consistent labels to repeated presentations of the stimuli. This effect occurs whether the child has relatively long or short exposure to the identifying phonetic cue, which was, in this case, the spectral envelope of a fricative consonant. Our findings rule out the possibility that the performance of children with dyslexia on the identification task is limited by a general difficulty processing brief auditory cues (Merzenich *et al.*, 1996), as well as the more recently posed hypothesis that they do not properly sample or integrate auditory information at the phonetic-cue scale (Goswami, 2011). Although we cannot make claims about temporal sampling or processing in all contexts, our results suggest that difficulty making categorical judgments about speech sounds is not primarily driven by temporal features of the stimulus. The benefit of increasing cue duration was roughly equal across the spectrum of reading abilities. If children with dyslexia were unable to form clear categories of speech sounds because they did not properly sample phonetic information (the spectral envelope), then we would have expected to find a significant interaction between stimulus duration and reading ability. This would occur because increasing the duration of phonetic cue availability would give children with dyslexia a greater chance of perceiving the cue, improving their performance on the labeling task relative to strong readers. Conversely, we might expect an interaction in the opposite direction if children with dyslexia were so profoundly impaired at processing the phonetic cue that these children derived little or no benefit from increased duration, while control participants did. In any case, the fact that we did not find evidence for such an interaction for any of the psychometric function parameters tested—including slope, asymptote, and the PC1 of these measures—argues that the well-established difficulty with phoneme identification in children with dyslexia is unlikely to be a consequence of impaired temporal processing.

There are several limitations to our study that must be noted. First, the nature of the task confounds our ability to tease apart cognitive processes that are specifically auditory and those that are domain general. While we are surely testing some aspects of auditory processing, phoneme categorization tasks are well known to be influenced by many non-sensory factors, including the manner in which the stimuli are presented and how participants are instructed (Cleary and Pisoni, 2001; Pisoni *et al.*, 1982; Pisoni and Lazarus, 1974). As other researchers in the area of dyslexia have noted, typical sensory processing is not sufficient to ensure categorical behavior (Vandermosten *et al.*, 2018), and while we have sought to make our task simple, we cannot wholly exclude the possibility that the cognitive load was greater for our participants with dyslexia. In particular, in order to avoid orthographic cues in the task, listeners were asked to choose between two differently colored snakes without text labels. We cannot be sure that this kind of abstract categorization was not *more* difficult for children with dyslexia than associating sounds with text. While we are reassured that we did not see a strong relationship between nonverbal IQ and task performance after controlling for reading skill, our nonverbal IQ measure, performance on a matrix reasoning task, may not be closely related to the cognitive demands of the auditory task.

A second limitation to our study is that children with dyslexia are a remarkably heterogeneous group: there is moderate comorbidity of dyslexia with both ADHD and language impairments, including speech sound disorders and specific language impairments (Germanò *et al.*, 2010; Pennington, 2006; Stevenson *et al.*, 2005). While none of our participants reported having been diagnosed with specific language impairment, we cannot be sure that they would not have met the criteria for diagnosis at some point in their lives. It remains possible that the relationship between reading skill and task performance is influenced by the presence of other developmental language impairments that we do not explore here. Our study can only speak to the specific case of ADHD. Specifically, we tested the effects of ADHD and nonverbal IQ and found that they were not significant predictors of task performance once reading skill was accounted for.

Despite these limitations, several conclusions can be drawn from our results. First, our results are incompatible with the “temporal sampling” hypothesis, which contends that individuals with dyslexia are unable to efficiently extract acoustic cues at the phonetic time scale because they do not have proper neural entrainment to the speech envelope. In the temporal sampling framework, weak entrainment to the speech envelope means a listener will sample phonetic information at sub-optimal phases in the speech signal. If this were the case, then increasing the duration that phonetic information is available to children with dyslexia should improve their performance on the phoneme labeling task by affording them more chances to glimpse the spectral envelope cue in our stimuli, whereas typically developing children would not be expected to improve much. However, our results suggest that children with dyslexia struggle to assign categorical labels regardless of how many “samples” of acoustic information may be available. Similarly, our results are at odds with the original formulation of the rapid temporal processing hypothesis (Tallal, 1980), which predicts that children with dyslexia will perform poorly on tasks with brief auditory cues but will perform more like typically developing children as auditory cue duration increases. We note that we did not test how perception changed with fricative durations exceeding 300 ms. While it is possible that perceptual effects could be observed by increasing the duration of phonetic cues into this range, both temporal theories of dyslexia (Goswami, 2011; Tallal, 1980) would still need to be amended to explain our current findings.

A second conclusion is that the relationship between reading skill and an index of categorical labeling–the shape of a psychometric function–is well modeled by a linear function. In other words, there is no obvious discontinuity in task performance between children who meet the criteria for dyslexia and children who do not. We found a similar result in our previous study of categorical labeling in 44 school-aged children (O'Brien *et al.*, 2018). This argues against interpreting children with dyslexia as a separate population from typical readers, at least with regard to the aspects of speech perception tested here. Although group-level statistics comparing a cohort of poor readers to unimpaired readers may reveal group differences, as we show here, the relationship between reading skill and our measure of categorical labeling is indeed continuous.

At this juncture, there have been nearly 40 studies of phoneme identification with reading-disabled children (for a summary of the literature, see Noordenbos and Serniclaes, 2015). There is no clear consensus on stimulus features that induce or remedy struggling readers' impairments with tasks that ostensibly probe categorical labeling: the deficit has been found for synthetic (Godfrey *et al.*, 1981; Manis *et al.*, 1997; Serniclaes *et al.*, 2001) and naturalistic speech, and for spectral (Johnson *et al.*, 2011; Nittrouer, 1999; O'Brien *et al.*, 2018; Steffens *et al.*, 1992), temporal (Bogliotti *et al.*, 2008; Breier *et al.*, 2001; Chiappe *et al.*, 2001; Hazan *et al.*, 2009), and spectrotemporal cues (Van Beinum *et al.*, 2005; Blomert and Mitterer, 2004; Boets *et al.*, 2011; Messaoud-Galusi *et al.*, 2011; Mody *et al.*, 1997). In the present study, we confirm that both brief and long stimulus presentations are difficult for children with dyslexia to categorize. Although some studies have shown group differences on certain continua but not others (Nittrouer, 1999; Vandermosten *et al.*, 2010; Vandermosten *et al.*, 2011), no reliable pattern seems to emerge for a specific acoustic feature that explains difficulties with phoneme categorization in children with dyslexia.

To make sense of these disparate results, it is important to remember that categorical labeling is a general feature of sensory perception, and the task design we employed draws upon working memory, statistical learning capacity, and auditory attention (Ramus and Ahissar, 2012). There has been growing interest in non-sensory explanations for why children with dyslexia tend to perform unusually on many tests of auditory processing, including their ability to form categories (Gabay and Holt, 2015), act as ideal observers (Ahissar *et al.*, 2006; Ziegler, 2008), and make use of statistics in sensory information (Gabay *et al.*, 2015; Vandermosten *et al.*, 2018). While we found no evidence that ADHD or nonverbal IQ explained task performance better than reading skill in our sample, these measures may not be effective probes of the cognitive processes involved in categorical learning and decision-making.

A final reason that we are inclined to suggest further research into general features of sensory decision-making in children with dyslexia, versus speech processing in particular, is that the relationship between the phoneme labeling task and speech perception in natural conditions may be complex. It is controversial whether categorical labeling is a necessary stage of analysis in speech processing (Cleary and Pisoni, 2001), and indeed there is evidence that *non-*categorical processing is part of word recognition in typical listeners (McMurray *et al.*, 2003; McMurray *et al.*, 2009; Toscano *et al.*, 2010). With few exceptions (e.g., McQueen *et al.*, 1999), there is little experimental evidence (that we know of) linking categorical perception at the scale of individual phonemes to speech understanding in naturalistic settings. Therefore, rather than treating phoneme identification tasks as probes of auditory acuity, or speech processing in everyday environments, it may be more fruitful to turn our attention to the domain-general functions that are tapped by such laboratory tasks. For now, the evidence from our study strongly suggests that differences in temporal processing cannot explain why some individuals with dyslexia behave differently in this experimental context.

## Footnotes

^1^ http://ReadingAndDyslexia.com (Last viewed July 9, 2019).

^2^ https://github.com/yeatmanlab/Parametric_speech_public/Stimuli (Last viewed July 9, 2019).

^3^ https://github.com/YeatmanLab/Parametric_speech_public (Last viewed July 9, 2019).
